# Editorial: Bayesian Estimation and Inference in Computational Anatomy and Neuroimaging: Methods and Applications

**DOI:** 10.3389/fnins.2019.00562

**Published:** 2019-05-29

**Authors:** Xiaoying Tang, Michael I. Miller

**Affiliations:** ^1^Department of Electrical and Electronic Engineering, Southern University of Science and Technology, Shenzhen, China; ^2^Center for Imaging Science, Johns Hopkins University, Baltimore, MD, United States; ^3^Department of Biomedical Engineering, Johns Hopkins University, Baltimore, MD, United States; ^4^Institute for Computational Medicine, Johns Hopkins University, Baltimore, MD, United States

**Keywords:** Bayesian estimation, computational anatomy, medical imaging, shape, prediction

This e-book brings together a total of nine studies focusing on imaging-based Bayesian estimation and computation. Computational tools were developed for various clinical purposes, including white matter (WM) lesion segmentation, statistical shape analysis, fiber tracking, anatomy coding, disease status and pathology detection and prediction, as well as functional connectivity analysis. Most studies focused on MRI whereas two analyzed respectively, OCT and PET. The investigations included a variety of populations, including healthy normal and patients with Multiple Sclerosis (MS), glaucoma, Alzheimer's disease (AD), Ataxia, primary progressive aphasia (PPA), Huntington's disease (HD), temporal lobe epilepsy (TLE), and Parkinson's disease (PD).

Jain et al. proposed a pipeline for segmenting two time point WM lesions in a joint expectation-maximization (EM) framework. The pipeline utilized two-modality MR images (a 3D T1-weighted image and a 3D FLAIR image). It modeled the lesion evolution between the two time points using a Gaussian mixture model and conducted simultaneous tissue and lesion segmentation in images from both time points. The model was optimized using a joint EM algorithm. The proposed pipeline was validated on two datasets, respectively involving 12 and 10 patients with MS.

Lee et al. conducted statistical shape analysis of the retinal nerve fiber layer (RNFL) and choroid in the framework of computational anatomy (CA), with OCT being used. A novel registration technique, namely functional shapes (fshape), was employed to match two retinas and to generate the mean of multiple retinas. In fshape, a diffeomorphism was obtained by a joint optimization of the surface geometry (the retinal surface) and functional signals mapped onto the surface (the retinal layer thickness). Point-wise analyses and visualizations were conducted using the fshape-derived diffeomorphisms. Using this technique, the authors successfully examined age-related and glaucoma-related spatial RNFL thickness patterns in 38 participants.

Dong et al. presented a method for fiber tracking in the Bayesian setting with geometric shape priors. The fiber tracts between regions of interest (ROIs) were initialized as Euclidean curves and then iteratively updated via deformations using gradients of a posterior energy. Estimations were performed using an energy function involving three components: the likelihood, the prior knowledge on the geometric shapes of fibers, and a roughness penalty term. The prior on the geometric shapes relied on atlas-based statistical shape models of fiber curves between ROIs. The proposed tractography methodology was evaluated on both simulated 2D data and 30 real 3D data from the Human Connectome Project (HCP).

Tward and Miller invented a strategy for anatomy coding using a Bayesian prior model. The entropy of an anatomy of interest was quantified as a function of code rate (number of bits). In this setting, the authors studied the shape of 12 subcortical structures of the human brain through diffeomorphic transformations relating each of them to a population-averaging and structure-specific template. A multivariate Gaussian prior model was trained using 650 MRI data from the Alzheimer's Disease Neuroimaging Initiative (ADNI). The authors found that at 1 mm all subcortical structures can be described with <35 bits, and at 1.5 mm all structures can be described with <12 bits.

Faria et al. explored the effectiveness of using MRI-based whole brain segmentations to extract key anatomical phenotypes for characterizing four neurodegenerative diseases (Ataxia, *n* = 16; HD, *n* = 52; AD, *n* = 66; and PPA, *n* = 50), all inducing brain atrophy. Homogeneous clinically-relevant phenotypes were successfully clustered. Using the structural quantification and simple linear classifiers, the authors were able to detect the four diseases with satisfactory accuracies. Moreover, the anatomical features automatically delivered by the classifiers agreed with the patterns of the disease pathologies.

Chiang et al. developed an integrative Bayesian prediction model to identify a brain's pathological status through a selection of fluoro-deoxyglucose PET imaging biomarkers. The proposed model was tested on 19 patients with TLE who subsequently underwent anterior temporal lobe resection. The proposed model successfully identified patient subgroups characterized by latent pathologies that associate differentially to clinical outcomes. It also yielded imaging biomarkers that describe the pathological states of the subjects. The proposed method was shown to achieve good accuracy in predicting post-surgical seizure recurrence.

Seiler and Holmes analyzed functional connectivity using two novel heteroscedasticity covariance models. The first model was low-dimensional, scaling linearly in the total number of brain parcellations. And the second model scaled quadratically. Both models were applied to the functional-resting fMRI data of 820 subjects from HCP, comparing connectivity between short and conventional sleepers. Stronger functional connectivity in short than conventional sleepers were found in brain regions that are consistent with previous findings.

Xue et al. proposed a Bayesian hierarchical model to predict disease status by incorporating information from both functional and structural brain imaging scans. Posterior probabilities were used to perform prediction, with the parameter estimations conducted on samples drawn from the joint posterior distribution using Markov Chain Monte Carlo methods. Predictions were conducted at both whole-brain and voxel levels, with the disease-related brain regions identified from the voxel-level prediction results. The proposed model was applied to a PD study, with key regions contributing to accurate prediction having been identified.

Tang et al. presented a fully-automated pipeline for generating subcortical and ventricular shapes from brain MR images. The proposed pipeline consisted of three key components: (1) automated structure segmentation; (2) study-specific shape template creation; (3) deformation-based shape filtering. The proposed pipeline was validated on two HD datasets, respectively involving 16 and 1,445 MRI scans. Another independent dataset, consisting of 15 atlas images and 20 testing images, was also used to quantitatively evaluate the proposed pipeline. High accuracy has been observed.

Together, these studies provide evidence for the power of Bayesian estimation theorem in imaging analysis. This e-book contains both methodology developments and scientific applications, with several imaging techniques having been involved (MRI, PET, and OCT). Three papers specifically lie in the CA framework, focusing on statistical shape analysis (Lee et al., Tward and Miller, and Tang et al.). These nine studies provide tools and examples for imaging-based computational analyses addressing various clinical questions and advances future research in this field.

## Author Contributions

XT wrote the paper and MM revised the manuscript critically for important intellectual content.

### Conflict of Interest Statement

MM owns an equal share in Anatomyworks LLC. The terms of this arrangement have been reviewed and approved by the Johns Hopkins University, in accordance with its conflict of interest policy. The remaining author declares that the research was conducted in the absence of any commercial or financial relationships that could be construed as a potential conflict of interest.

